# Investigation of the relationship between ocular sarcoidosis and dry eye

**DOI:** 10.1038/s41598-022-07435-6

**Published:** 2022-03-02

**Authors:** Takanori Aoki, Norihiko Yokoi, Kenji Nagata, Hideto Deguchi, Yuki Sekiyama, Chie Sotozono

**Affiliations:** Department of Ophthalmology, Kyoto Prefectural University of Medicine, 465 Kajii-cho, Hirokoji-agaru, Kawaramachi-dori, Kamigyo-ku, Kyoto, 602-0841 Japan

**Keywords:** Eye diseases, Eye manifestations

## Abstract

A relationship between ocular sarcoidosis (OcSar) and dry eye (DE) has previously been reported. Here we investigated the tear function in OcSar, and the other uveitis, Vogt–Koyanagi–Harada disease (VKH), to elucidate the association between OcSar and DE. This study involved 27 eyes of 27 female OcSar patients [mean age: 67.3 ± 13.5 (SD) years], 18 eyes of 18 female VKH patients (53.1 ± 14.1 years), and 17 eyes of 17 female healthy control subjects (59.7 ± 9.6 years). In all examined eyes, tear meniscus height (TMH, mm), fluorescein breakup time (FBUT, s), scoring of keratoconjunctival epithelial damage (ED), and the Schirmer 1 test (ST1, mm/5 min) were analyzed. In the OcSar patients, VKH patients, and control subjects, the respective TMH, FBUT, ED, and ST1 values (mean ± SD) were 0.26 ± 0.10, 0.24 ± 0.09, and 0.24 ± 0.08, 4.3 ± 2.5, 7.3 ± 2.9, and 6.3 ± 3.1, 1.1 ± 1.1, 0.7 ± 0.8, and 0.1 ± 0.3, and 12.9 ± 10.5, 21.5 ± 12.1, and 24.3 ± 10.5. Statistically, the ST1 values were significantly lower in the OcSar patients than in the VKH patients (*P* = 0.004) and control subjects (*P* = 0.001). ST1 findings revealed that the neural reflex arc and lacrimal gland system, which attenuate the vicious cycle between the tear film and ocular surface epithelium in DE, are significantly impaired in OcSar cases, thus indicating a possible association between OcSar and DE.

## Introduction

Sarcoidosis is a systemic disease in which noncaseating granuloma lesions distribute in the lung, heart, eyes, skin, cardiac, spleen, etc.^1^. In the Japanese population, sarcoidosis commonly affects the lungs, such as bilateral hilar lymphadenopathy (BHL) (86% to 98.5% of cases), followed by the ocular sarcoidosis (OcSar) (55% to 68% of cases)^2,3^. OcSar can involve any part of the eye and its adnexal tissues, and may cause uveitis [i.e., uveitis sarcoidosis (UvSar)], lacrimal gland swelling [i.e., lacrimal sarcoidosis (LaSar)], episcleritis, eyelid abnormalities, conjunctival granuloma, and optic neuropathy^4^. Most OcSar cases are UvSar, which is known to have the features of granulomatous inflammation. LaSar is known to be associated with bilateral eyelid swelling and dry eye (DE), mainly in young females, and is sometimes accompanied by various systemic complications such as BHL or parotid swelling. Histopathologically, LaSar is characterized by noncaseating granuloma in the lacrimal gland^5^. Reportedly, the incidence of LaSar in OcSar ranges from 4.2 to 15.8%^3,6^, and the majority of sarcoidosis diagnosed at the orbital region is LaSar. However, the incidence of LaSar among all sarcoidosis types is very rare (1.7% to 2.4% of all sarcoidosis cases)^3,5–7^.

A relationship between OcSar (mainly UvSar) and DE, keratoconjunctivitis sicca, and Sjögren’s syndrome (SS) has previously been reported^8–10^. In general, it is thought that DE in OcSar is primarily related to lacrimal gland inflammation/infiltration of inflammatory cells that results in decreased tear production^11^. In LaSar cases, the inflammation reportedly occurs primarily in the main lacrimal gland, thus indicating a relationship between LaSar and DE^4^. However, due to the limited number of reported findings, the exact relationship has yet to be fully clarified.

According to the International Dry Eye WorkShop II (DEWS II) definition, DE is classified into two categories, i.e., aqueous tear deficient DE and evaporative DE^12^, yet the conditions of those two categories often reported to be mixed, so that aqueous tear deficient DE and evaporative DE may exist as combined rather than separate entities^13^. However, according to the Asia Dry Eye Society (ADES) classification, DE is classified into aqueous deficient DE, decreased wettability DE, and increased evaporation DE, with the latter two being classified as short-breakup-time (BUT)-type DE. According to ADES, in all types of DE, tear film instability plays a central and common role in their pathophysiology^14^, and various risk factors can cause instability of the tear film, thus leading to a vicious cycle between the tear film and the ocular surface epithelium that can result in a variety of symptoms. Regarding the categorization of the risk factors for DE through DEWS II classification, the relationship between DE and OS is not conclusive^15^.

Although the previous study showed pathologically the inflammation in the main or accessory lacrimal glands of sarcoidosis patients, the specific tear secretion function in those patients remains unclear. Therefore, the purpose of this study was to elucidate the possible association between OcSar (UvSar + LaSar) and DE, and DE subtype, by comparing with both Vogt–Koyanagi–Harada disease (VKH) as an uveitis control and healthy subjects as a normal control via the investigation of the tear functions and ocular surface epithelial abnormalities.

## Results

**Figure 1:**
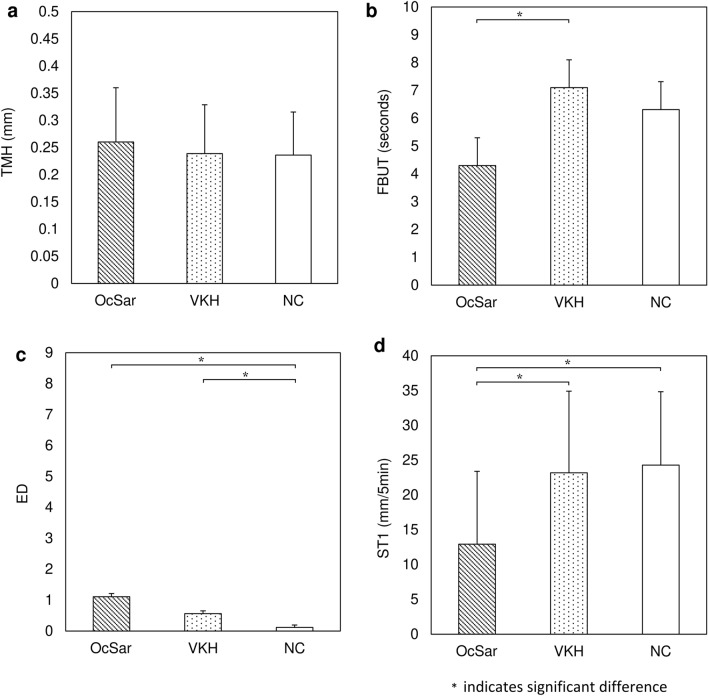
(**a**) Comparison of tear meniscus height (TMH, mm) between the ocular sarcoidosis (OcSar) group (0.26 ± 0.10 mm), Vogt–Koyanagi–Harada disease (VKH) group (0.24 ± 0.09 mm), and normal control (NC) group (0.24 ± 0.08 mm) (all values are mean ± SD). No statistical difference was found. (**b**) Comparison of fluorescein breakup time (FBUT, seconds) between the OcSar group (4.3 ± 2.5 s), VKH group (7.3 ± 2.9 s) and OcSar group (6.3 ± 3.1 s) (all values are mean ± SD). Significant differences were found between the one of 3 groups [*P* = 0.002 (OcSar vs. VKH), *P* = 0.057 (OcSar vs. NC), and *P* = 0.527 (VKH vs. NC)]. (**c**) Comparison of keratoconjunctival epithelial damage (ED) between the OcSar group (1.1 ± 1.1), VKH group (0.7 ± 0.8), and NC group (0.1 ± 0.3) (all values are mean ± SD). There were significant differences between the two of 3 groups [*P* = 0.358 (OcSar vs. VKH), *P* = 0.001 (OcSar vs. NC), and *P* = 0.040 (VKH vs. NC)]. (**d**) Comparison of the Schirmer 1 test results (ST1, mm/5 min) between the OcSar group (12.9 ± 10.5 mm), VKH group (21.5 ± 12.1 mm), and NC group (24.3 ± 10.5 mm) (all values are mean ± SD). The ST1 values in the OcSar group were significantly lower than those in the VKH group and NC group [*P* = 0.004 (OcSar vs. VKH), *P* = 0.001 (OcSar vs. VKH)].

This retrospective study included 27 eyes of 27 patients in the OcSar group, 18 eyes of 18 patients in the VKH group, and 17 eyes of 17 subjects in the normal control (NC) group. In the OcSar group there were 26 UvSar patients and only 1 LaSar patient. The demographic data obtained from the subjects, including age, measurement of tear meniscus height (TMH), measurement of fluorescein BUT (FBUT), scoring of keratoconjunctival epithelial damage (ED), the Schirmer 1 test (ST1), and the rate of subjects with an ST1 of ≤ 5 mm/5 min is shown in Table 1, and the statistical analysis findings among each group for age, TMH, FBUT, ED, ST1, and the rate of patients with an ST1 of ≤ 5 mm/5 min is shown in Table 2. The mean patient age was 67.3 ± 13.5 (mean ± SD) years (range 42 to 86 years) in the OcSar group, 53.1 ± 14.1 years (range 21 to 81 years) in the VKH group, and 59.7 ± 9.6 years (range 45 to 77 years) in the NC group. No statistically significant difference was found in the comparison of TMH values between the OcSar group (0.26 ± 0.10 mm), VKH group (0.24 ± 0.09 mm), and NC group (0.24 ± 0.08 mm) [*P* = 0.687 (OcSar vs. VKH), *P* = 0.670 (OcSar vs. NC), and *P* = 0.999 (VKH vs. NC)] (Fig. 1a). The mean FBUT in the OcSar group (4.3 ± 2.5 s) was significantly lower than that in the VKH group (7.3 ± 2.9 s) (*P* = 0.002), yet no significant difference in mean FBUT was found between the OcSar group (4.3 ± 2.5 s) and the NC group (6.3 ± 3.1 s) (*P* = 0.057) (Fig. 1b). There was no significant difference in the mean ED value between the OcSar group (1.1 ± 1.1) and the VKH group (0.7 ± 0.8) (*P* = 0.358). The mean ED value in the OcSar group (1.1 ± 1.1) was significantly higher than that in the NC group (0.1 ± 0.3) (*P* = 0.001), and the mean ED value in the VKH group was significantly lower than that in the NC group (*P* = 0.040) (Fig. 1c). The mean ST1 value in the OcSar group (12.9 ± 10.5 mm) was significantly lower than that in the VKH group (21.5 ± 12.1 mm) and NC group (24.3 ± 10.5 mm) [*P* = 0.004 (OcSar vs. VKH), *P* = 0.001 (OcSar vs. VKH)] (Fig. 1d). The rate of the cases with ST1 values being 5 mm or less was 8/27 (29.6%) in the OcSar group, 1/18 (5.6%) in the VKH group, and 0/17 (0%) in the NC group. The rate of cases with ST1 values of ≤ 5 mm was analyzed among 2 groups each by Fisher’s exact test. The rate of cases in each group with ST1 values of ≤ 5 mm was significantly higher in the OcSar group than in the NC group (*P* = 0.016). There was no significant difference in the rate of cases with ST1 values of ≤ 5 mm between 2 of the 3 groups [*P* = 0.064 (OcSar vs. VKH), *P* = 1.000 (VKH vs. NC)].

**Table 1:** Demographic data of the ocular sarcoidosis (OcSar) group, Vogt–Koyanagi–Harada disease (VKH) group, and normal control (NC) group.

	OcSar (UvSar + LaSar)	LaSar	VKH	NC
Number of eyes	27	1	18	17
Mean age (years)	67.3 ± 13.5	43	53.1 ± 14.1	59.7 ± 9.6
TMH (mm)	0.26 ± 0.10	0.25	0.24 ± 0.09	0.24 ± 0.08
FBUT (s)	4.3 ± 2.5	8.3	7.3 ± 2.9	6.3 ± 3.1
ED	1.1 ± 1.1	0	0.7 ± 0.8	0.1 ± 0.3
ST1 (mm/5 min)	12.9 ± 10.5	0	21.5 ± 12.1	24.3 ± 10.5
Rate of patients with ST1 ≤ 5 mm/5 min	8/27 (29.6%)	1/1 (100%)	1/18 (5.6%)	0/17 (0%)

Values are mean ± SD.

*UvSar* uveitis sarcoidosis, *LaSar* lacrimal sarcoidosis, *FBUT* fluorescein breakup time, *TMH* tear meniscus height, *ED* keratoconjunctival epithelium disorder, *ST1* Schirmer 1 test.

**Table 2:** Statistical comparison of the demographic data between the OcSar group, VKH group, and NC group.

	OcSar vs. VKH	OcSar vs. NC	VKH vs. NC	
Age	0.002*	0.144	0.279	Tukey–Kramer
TMH	0.687	0.670	0.999	Tukey–Kramer
FBUT	0.002*	0.057	0.527	Tukey–Kramer
ED	0.358	0.001*	0.040*	Steel–Dwass
ST1	0.004*	0.001*	0.733	Tukey–Kramer
Rate of patients with ST1 ≤ 5 mm/5 min	0.064	0.016*	1.000	Fisher’s exact test

*OcSar* ocular sarcoidosis, *VKH* Vogt-Koyanagi-Harada disease, *NC* normal control, *TMH* tear meniscus height, *FBUT* fluorescein breakup time, *ED* keratoconjunctival epithelial damage, *ST1* Schirmer 1 test.

*Statistically significant difference.

## Discussion

The findings in this study revealed that the ST1 values of the OcSar group were significantly less than those of the VKH group and NC group [*P* = 0.004 (OcSar vs. VKH), *P* = 0.001 (OcSar vs. NC)]. The rate of cases with ST1 values of ≤ 5 mm was higher in the OcSar group than in NC group [*P* = 0.016 (OcSar vs. NC)]. Although there was no significant difference in the rate of cases with ST1 values of ≤ 5 mm between the OcSar group and the VKH group, there was a trend of the rate being higher in OcSar than in VKH [*P* = 0.064 (OcSar vs. VKH)]. However, there was no significant difference in TMH values among the 3 groups. As for the FBUT values, the values in the OcSar group were significantly lower than those in the VKH group (*P* = 0.002). As for the ED values, there were significant differences between the two of 3 groups [*P* = 0.358 (OcSar vs. VKH), *P* = 0.001 (OcSar vs. NC), and *P* = 0.040 (VKH vs. NC)]. However, the overall ED score was minimal in all 3 groups (0.1–1.1; 9 points maximum), thus suggesting less meaningful results. In addition, in the one LaSar case in the OcSar group, FBUT was 8.3 s, ED was 0, and the ST1 value was 0 mm/5 min. Those results indicate that there was lacrimal gland dysfunction in the OcSar group, although the tear volume at the ocular surface was retained at the normal level, this possibly being an explanation for the minimal involvement of ED in the OcSar group.

ST1 values are thought to reflect both of the reflexively secreted tear volume and basal tear volume^16,17^. However, from a physiological point of view, reflex tear secretion assessed by ST1 is thought to more reflect the function of the main lacrimal gland than that of accessory lacrimal glands^11,18^. Conversely, TMH is thought to be associated with basal tear volume^11^ which would mainly contribute to the precorneal tear film stability assessed by FBUT, because TMH is proportional to tear meniscus radius, which determines the thickness of aqueous layer of the precorneal tear film.

In the few reported studies on the relationship between UvSar and ST1, 20–64% of UvSar cases reportedly exhibited 10 mm/5 min or less in the ST1, and the one combined case of UvSar and LaSar reportedly had DE with severe corneal conjunctival damage^10,19^. Table 3 summarizes the previous reports in which the relationship between LaSar cases and ST1 was demonstrated, and 4 of 6 reported cases showed 5 mm/5 min or less in the ST1^20–25^. Our findings are compatible with those in the previous studies, in which OcSar and LaSar showed that ST1 is more decreased than those from VKH and NC, thus indicating lacrimal gland dysfunction, possibly attributable to that of main lacrimal glands. However, it should be noted that our study first indicated that the basal tear secretion in OcSar, as assessed by TMH, was equivalent to those in VKH and the NC, which may further indicate that the function of basal tear secretion, or the function of accessory lacrimal glands, is maintained in OcSar. It is interesting that the present results are compatible with the previous reports that inflammation associated with OcSar is demonstrated in the main lacrimal gland, not in the accessory lacrimal glands^4,11^. Considering that the main lacrimal glands are the components that constitute the neural reflex-loop and lacrimal gland system, and that the background disease and/or conditions that are associated with the dysfunction of the neural reflex loop comprising the sensory nerve and parasympathetic nerve were excluded from our study, our findings seem to indicate that dysfunction of the main lacrimal gland may be involved in OcSar. Our findings demonstrate that OcSar is likely to present with short BUT-type DE (FBUT in OcSar; 4.3 ± 2.5). However, our finding that the TMH remained normal, in spite of the decreased ST1, may suggest a predisposition to aqueous deficient DE.

**Table 3:** Cases of sarcoidosis with lacrimal gland involvement.

Case	Author	Year	Age	Gender	ST1	Involvement
1	Genma et al.^20^	1986	42	F	1	LaSar, parotid glands, bone, skin
2	Hegab et al.^21^	1988	16	F	1	LaSar, conjunctiva
3	Yoshioka et al.^22^	2006	25	M	≤ 5	LaSar, BHL, parotid glands, skin, pituitary gland
4	Kawashiri et al.^23^	2015	60	M	> 5	LaSar, BHL
5	Kobak et al.^24^	2015	24	F	> 5	LaSar, BHL
6	Bingol Kiziltunc et al.^25^	2017	20	F	1	LaSar, BHL, parotid glands, submandibular glands, lymph nodes

*ST1* the Schirmer 1 test, *LaSar* Lacrimal sarcoidosis, *BHL* bilateral hilar lymphadenopathy.

It has been reported that the clinical diagnosis of UvSar can be done through the bilateral conjunctival biopsy and noncaseating granuloma in the conjunctiva that is demonstrated in 38% to 71% of UvSar cases^26,27^. The presence of noncaseating granuloma in the conjunctiva suggests that inflammation similar to that in the lacrimal gland may be involved in the conjunctiva in UvSar. This relationship may be similar to that in cases with SS, in which both the conjunctiva and lacrimal gland are similarly affected by the inflammation associated with SS, and conjunctival inflammation in SS may partly contribute to the manifestation of tear film instability (shorter FBUT) and ED^28^.

In healthy eyes, there is a good relationship between stable tear film and healthy corneal epithelium, each mutually maintaining the other. However, in DE cases, various risk factors cause unstable tear film or ocular surface epithelial wettability, which results in a vicious cycle between the tear film and corneal surface epithelium. In such cases, the auto-repair system (neural reflex loop—lacrimal gland system) works to repair the vicious circle and attempts to restore the healthy relationship^29^. In this present study, this system, as evaluated by ST1, was found to be significantly impaired in OcSar, while TMH and FBUT not being reflected upon^30^. Accordingly, it is possible that in OcSar, DE is manifested only in the situation when neural reflex loop and lacrimal gland system is used. For example, steroid eye drops and anti-glaucoma eye drops, respectively, are often used for the treatment of OcSar with uveitis and secondary glaucoma. However, those eye drops potentially have a risk to deteriorate not only the tear film, but also corneal surface epithelium, both leading to DE via the establishment of a vicious cycle between the tear film and corneal surface epithelium^31,32^. In such cases, the neural reflex loop and lacrimal gland system may work to attenuate the vicious cycle and attempt to restore the healthy condition. From this point of view, since this system is impaired in OcSar, it is important to keep it in mind that OcSar is more susceptible to the insult into the tear film and/or surface epithelium either of which becomes a trigger to the manifestation of DE. In this study, the rate of patients who used eye drops including benzalkonium chloride (BAC) was 6/27 (22.2%) in the OcSar group, 3/18 (16.7%) in the VKH group, and 0/17 (0%) in the NC group. There was no significant difference between the rate of patients using eye drops including BAC in all 3 groups [*P* = 0.721 (OcSar vs. VKH), *P* = 0.067 (OcSar vs. NC), *P* = 0.229 (VKH vs. NC)]. Moreover, the rate of patients who used corticosteroid eye drops was 21/27 (77.8%) in the OcSar group, 5/18 (27.8%) in the VKH group, and 0/17 (0%) in the NC group. A significant difference in the rate of patients using corticosteroid eye drops was found between all 3 groups [*P* = 0.002 (OcSar vs. VKH), *P* < 0.001 (OcSar vs. NC), *P* = 0.046 (VKH vs. NC)]. Although it is possible that glaucoma eye drops including BAC may have affected DE worse and that corticosteroid eye drops may have affected DE better, the counteracting effect on DE of each ingredient may have produced no effect.

It should be noted that this study did have some limitations, as it was a retrospective study involving a small number of subjects. First, there was a difference in the average patient age between the OcSar group and the VKH group, probably due to differences in the age at which each disease onset occurred. Second, since the uveitis patients required treatment with corticosteroid eye drops and glaucoma eye drops, the background of the eye drops administered among the 3 groups was not unified. However, with those study limitations set aside, it should be noted that there have been few reports on the assessment of tear function for OcSar, we theorize that the findings in this present study will open new insights into the possible relationship between OcSar and LaSar, as well as to the DE type associated with those clinical entities via the interpretation of the several DE tests, including ST1, TMH, and BUT.

## Conclusions

In this study, the ST1 findings, not the TMH and BUT findings, revealed that the neural reflex loop and lacrimal gland system, which works to attenuate the vicious cycle between tear film and ocular surface epithelium in DE, are significantly impaired in OcSar by comparison with VKH as an uveitis control and healthy subjects. Thus, OcSar is possibly associated with DE via impairment of the neural reflex loop and lacrimal gland system.

## Methods

This retrospective cross-sectional study was conducted from April 2017 to September 2020 at Kyoto Prefectural University of Medicine, Kyoto, Japan. The following three groups of subjects were enrolled in this study: (1) OcSar patients under treatment (OcSar group), (2) VKH patients with uveitis as an uveitis control (VKH group), and (3) healthy normal subjects as a normal control (NC group). Two methods were used to make a definitive diagnosis of OcSar, histopathological diagnosis and clinical diagnosis based on the International Workshop on Ocular Sarcoidosis criteria^33^. VKH was diagnosed based on the VKH diagnostic criteria, and steroid pulse therapy had been performed in all cases^34^.

All enrolled subjects underwent the following four examinations in successive order: (1) measurement of tear meniscus height (TMH), (2) measurement of fluorescein BUT (FBUT), (3) scoring of keratoconjunctival epithelial damage (ED), and (4) the Schirmer 1 test (ST1). TMH and FBUT were measured via a less-invasive staining method. Briefly, a fluorescein strip (AYUMI Pharmaceutical Corporation, Tokyo, Japan) was vigorously shaken after instillation of two saline eye drops, with the central top of the strip then being gently touched to the central lower lid margin in order to avoid any increase of tear volume^35^. Immediately after staining, a photograph was taken using a slit-lamp biomicroscope (Slit lamp SL-D7; Topcon Corporation, Tokyo, Japan and Digital Camera D7000; Nikon Corporation, Tokyo, Japan) at 24-times magnification, and TMH (mm) was then measured by use of a GNU Image Manipulation Program micro ruler [GIMP Organization (www.gimp.org)] at the central lower tear meniscus. This method was adopted to this study after confirming that TMH was statistically unchanged between before and after the staining of tears with fluorescein^36^. After the photograph was taken, the patient was verbally instructed to open the eye and keep the eye open after gentle closing of the eye^35^, and FBUT (seconds) was measured 3 times, and then averaged. If the FBUT was more than 10 s, it was described as 10 s, because keeping an eye open for more than 10 s may result in reflex tearing which interferes with each following FBUT measurement. ED was evaluated by use of the van Bijsterveld score (from a minimum of 0 to a maximum of 9)^37^. At more than 10 min after the FBUT and ED evaluations were performed, ST1 was performed to measure reflex tear secretion over a 5-min period without anesthesia under natural blinking. This study involved only female subjects, and in all subjects, right eyes were selected for examination.

### Statistical analysis

JMP^®^ Pro ver. 14.0.0 statistics software (SAS, Cary, NC, USA) was used for statistical analyses. The Tukey–Kramer method and the Steel–Dwass method were used to compare continuous variables, and Fisher’s exact test was used to compare categorical variables between the treatment groups. A P-value of < 0.05 was considered statistically significant.

### Ethical standards

The study was approved by the Review Board of Kyoto Prefectural University of Medicine (#ERB-C-138). The study was performed in accordance with the ethical standards as laid down in the 1964 Declaration of Helsinki and its later amendments or comparable ethical standards. And informed consent was obtained from all individual participants included in the study.

## Abbreviations

OcSar: Ocular sarcoidosis
UvSar: Uveitis sarcoidosis
LaSar: Lacrimal sarcoidosis
VKH: Vogt–Koyanagi–Harada disease
NC: Normal control
DE: Dry eye
SS: Sjögren’s syndrome
BHL: Bilateral hilar lymphadenopathy
TMH: Tear meniscus height
FBUT: Fluorescein breakup time
ED: Scoring of keratoconjunctival epithelial damage
ST1: Schirmer 1 test
